# Exercise‐induced calf muscle hyperemia: Rapid mapping of magnetic resonance imaging using deep learning approach

**DOI:** 10.14814/phy2.14563

**Published:** 2020-08-18

**Authors:** Jeff L. Zhang, Christopher C. Conlin, Xiaowan Li, Gwenael Layec, Ken Chang, Jayashree Kalpathy‐Cramer, Vivian S. Lee

**Affiliations:** ^1^ Athinoula A. Martinos Center for Biomedical Imaging Department of Radiology Massachusetts General Hospital Boston MA USA; ^2^ Department of Radiology and Imaging Sciences University of Utah Salt Lake City UT USA; ^3^ Department of Kinesiology University of Massachusetts Amherst MA USA; ^4^ Institute for Applied Life Sciences University of Massachusetts Amherst MA USA; ^5^ MGH and BWH Center for Clinical Data Science Massachusetts General Hospital Boston MA USA; ^6^ Verily Life Sciences Cambridge MA USA

**Keywords:** deep learning, magnetic resonance imaging, muscle hyperemia, plantar flexion, tracer kinetic analysis

## Abstract

Exercise‐induced hyperemia in calf muscles was recently shown to be quantifiable with high‐resolution magnetic resonance imaging (MRI). However, processing of the MRI data to obtain muscle‐perfusion maps is time‐consuming. This study proposes to substantially accelerate the mapping of muscle perfusion using a deep‐learning method called artificial neural network (NN). Forty‐eight MRI scans were acquired from 21 healthy subjects and patients with peripheral artery disease (PAD). For optimal training of NN, different training‐data sets were compared, investigating the effect of data diversity and reference perfusion accuracy. Reference perfusion was estimated by tracer kinetic model fitting initialized with multiple values (multigrid model fitting). Result: The NN method was much faster than tracer kinetic model fitting. To generate a perfusion map of matrix 128 × 128 on a same computer, multigrid model fitting took about 80 min, single‐grid or regular model fitting about 3 min, while the NN method took about 1 s. Compared to the reference values, NN trained with a diverse group gave estimates with mean absolute error (MAE) of 15.9 ml/min/100g and correlation coefficient (R) of 0.949, significantly more accurate than regular model fitting (MAE 22.3 ml/min/100g, R 0.889, *p* < .001). Conclusion: the NN method enables rapid perfusion mapping, and if properly trained, estimates perfusion with accuracy comparable to multigrid model fitting.

## Graphical abstract

A recent study reported the potential of using DCE MRI method to map muscle perfusion after stimulation of exercise. This current study proposed a deep learning method to enable rapid processing of the data so that perfusion maps can be obtained online.

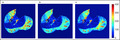

## 1 INTRODUCTION

Mapping of tissue perfusion can be achieved with dynamic contrast‐enhanced magnetic resonance imaging (DCE MRI) in many applications, such as cancer (Bernstein, Homer, & West, 2014; Mazaheri, Akin, & Hricak, 2017) and kidneys (Lee et al., 2001; Vivier, Storey, & Rusinek, 2011; Xie, Layton, & Wang, 2016; Yamamoto, Zhang, & Rusinek, 2011). With high spatial resolution of modern MRI techniques, maps of tissue perfusion can reveal and quantify overall and regional tissue heterogeneity and help improve the diagnosis of a range of pathological conditions. Recently, exercise stimulation was successfully incorporated into MRI exams so that calf‐muscle hyperemia can be quantitatively assessed with low‐dose DCE MRI ( Zhang, Layec, & Hanrahan, 2019). The obtained calf‐muscle perfusion maps could provide valuable information on the functional status (such as walking performance) of the muscles that is impaired in elderly subjects or patients with peripheral artery disease (PAD) (Isbell, Epstein, & Zhong, 2007; Pollak, Meyer, & Epstein, 2012; Zhang et al., 2019). Other experiments of exercise physiology (Andersen & Saltin, 1985; Joyner & Casey, 2015) may also benefit from the MRI technique because to our knowledge it is the only noninvasive clinical method available that reliably maps real‐time muscle hyperemia.

One major challenge with the muscle‐perfusion measurement is its time‐consuming image processing. Quantification of DCE MRI data relies on tracer kinetic analysis, in which tissue contrast enhancement (TC) is deconvolved from an arterial input function (AIF) to estimate tissue perfusion. AIF is sampled from an arterial region in the images. Deconvolution is typically implemented by iteratively optimizing the parameter values of an impulse retention function, so that its convolution with AIF fits the measured TC (Koh, Cheong, Hou, & Soh, 2003; Lee, Rusinek, & Bokacheva, 2007; St Lawrence & Lee, 1998). For perfusion mapping, the deconvolution is done for each voxel individually, a time‐consuming process that makes real‐time implementation unrealistic. In addition, the optimization may converge on wrong solutions, particularly for optimization problems with multiple local minima. Since it is not feasible to check the fitting outcome for every voxel in an image manually, to avoid misfit it is necessary to carefully select the initial value and searching range for the model parameters for each specific application. Proper implementation of the entire procedure can be challenging even for an experienced operator.

Artificial neural networks (NN) may improve the speed and accuracy of the perfusion quantification of DCE MRI data. NN decomposes complex operations into multiple layers, each consisting of a number of nodes. Each node receives input from nodes in the previous layer, performs simple transforms, and passes the output to the nodes in next layer. The many layers in a neural network allows learning of hierarchical representations of the input data that, with proper training, can be used to perform complex operations (LeCun, Bengio, & Hinton, 2015). With only simple arithmetic transforms, an NN model can be easily programmed on any computer platform. A NN has the additional benefit of mitigating convergence issues of model fitting as well as nearly instantaneous inference. The promise of the method has been demonstrated in recent study with patients of mild ischemic stroke (Ulas, Das, & Thrippleton, 2018). DCE MRI of skeletal muscles often consists of multiple scans stimulated by exercise of different intensities (Zhang et al., 2019). Real‐time perfusion mapping for an earlier scan may provide important information that guides precise prescription of exercise stimuli for later scans.

In this study, we tested the feasibility of implementing the NN method for quantifying exercise‐stimulated perfusion of calf muscles from DCE MRI. Unlike other organs, muscle perfusion can vary dramatically with exercise intensity, and pathologic conditions such as PAD or aging can further increase the variation range. To accurately estimate perfusion across such wide range using the NN method, we propose to increase the diversity of the training data, by including data from different subject populations and exercise intensities. We also tested the importance of reference value accuracy for NN training.

## 2 MATERIALS AND METHODS

This section first describes the exercise‐stimulated DCE MRI scans, including data acquisition and processing. We then introduce how we implemented the NN approach for estimating muscle perfusion from DCE MRI data. Furthermore, the network was trained by different strategies, including human data of different diversities, and the trained networks were compared by assessing performance on a hold‐out test set of human subjects.

### 2.1 Exercise‐stimulated DCE MRI for calf muscles

In this IRB‐approved study, 21 subjects signed written informed consent and participated in exercise‐stimulated DCE MRI scans. Thirteen of the subjects were young healthy volunteers (28 ± 6 years, eight females), five were elderly healthy volunteers (63 ± 4 years, one female), and three were patients with PAD (62 ± 1 years, one female, ankle‐brachial index 0.81 ± 0.09). Prior to the start of MRI, calf muscles were stimulated by plantar flexion of a single leg that pushed a loaded pedal at a frequency of 1 Hz. The exercise protocols included constant load of 4 lbs, 8 lbs, or 16 lbs for 3 min, and exercise to exhaustion. The exhaustion exercise started with a load of 2 lbs, and increased by 2 lbs every minute until subjects reported exhaustion. Each of the 21 subjects was separately scanned after two or four exercise protocols, and we collected a total of 48 MRI scans. The data were divided into two groups. “The diverse group” consisted of three young healthy subjects, five elderly healthy subjects, and three PAD patients, with overall 28 DCE MRI datasets (Table 1). As summarized in Table 1, eight of the subjects (20 datasets) were used for a primary training of NN, and the remaining three subjects (8 datasets) for network testing. “The homogeneous group” consisted of 10 young healthy subjects (20 datasets), each subject being scanned twice with stimulation by the same 8‐lbs exercise. The homogeneous group was also used for NN training for comparison to the NN training by the diverse group.

**Table 1:** Group composition of the diverse group that was used for NN training and testing

Subjects	Exercises to stimulate MRI	Data for training (subject #/scan #)	Data for testing (subject #/scan #)
three young healthy	4, 8, 16 lbs and exhaustion	2/8	1/4
five elderly healthy	4 lbs and exhaustion	4/8	1/2
three PAD patients	4 lbs and exhaustion	2/4	1/2

For MRI scans, subjects were positioned supine and feet‐first in a 3‐T MRI scanner (TimTrio; Siemens), with one calf wrapped in a 4‐channel flex receiver coil. The extremity scanned was either the right calf for the healthy volunteers or the more symptomatic calf for the patients. In the imaging position, subjects performed the plantar flexion protocol as described above. Immediately at the end of the exercise, 0.05 mmol/kg gadoteridol (Prohance; Bracco) was injected intravenously at a rate of 5 ml/s, followed by 20 ml saline injected with the same rate. Dynamic T_1_‐weighted images were acquired for one axial slice on the level of the maximal cross‐section area of the calf and one axial slice on the knee level with two‐dimensional saturation‐recovery turboFLASH: delay time 300 ms, repetition time (TR) 527 ms, echo time (TE) 1.42 ms, flip angle 15°, slice thickness 10 mm, matrix 128 × 128, field of view (FOV) 160 × 160 mm, temporal resolution 1 s/frame. The dynamic imaging lasted for 4 min, and the images from the first 40 s were used for perfusion quantification. To quantify tracer concentration from the MR signals, proton density was measured for the same slice using the same sequence with TR of 4,000 ms. For each dataset, an experienced user manually drew a region of interest (ROI) to exclude voxels of subcutaneous fat and background. AIF was manually sampled in the dynamic images from the peroneal artery, anterior tibial artery, and/or posterior tibial artery where partial volume artifact was absent. Both the sampled arterial signals and the tissue‐voxel signals were converted to tracer concentrations, denoted as AIF and TC respectively, based on a formula of T_1_‐shortening effect of gadolinium contrast (Zhang et al., 2019).

### 2.2 Tracer kinetic model fitting to estimate perfusion

**Figure 1:**
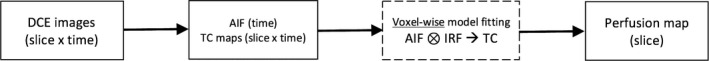
Schematic diagram of perfusion mapping in dynamic contrast enhanced magnetic resonance imaging (DCE‐MRI). In the acquired dynamic images, an arterial input function (AIF) is manually sampled from a large arterial region, and all the images are converted to maps of tissue contrast enhancement (TC). For each voxel of tissue, parameter values of impulse retention function (IRF) are optimized to fit the convolution of AIF and IRF to the voxel's TC. Tissue perfusion is a parameter of IRF. Completion of the model fitting for all voxels in a slice would result in a perfusion map. Dimensions of each data are shown in parentheses. For example, AIF is a one‐dimension vector as a function of time, and perfusion map has the same dimension as image slice

Tracer kinetic analysis is the established approach for quantifying tissue perfusion from DCE MRI data (Koh et al., 2003; Lee et al., 2007; Tofts, 1997). For the analysis, a model of impulse retention function (IRF) is predefined to characterize the response of the tissue of interest after a unit impulse input from the feeding artery. Tissue perfusion is one of the model's parameters. To estimate the parameter values from the measured AIF and TC, nonlinear least square optimization is used to minimize the residue between the measured TC and the model prediction (convolution between IRF and the measured AIF). In this study, we used an adiabatic tissue homogeneity model (Dennis Cheong, Tchoyoson Lim, & Koh, 2004) for IRF,

(1) $$IRF\left(t\right)=\left\{\begin{array}{cc} 0 & t<t_{0}\\ F & t<t_{0}+\min TT\\ F\cdot E\cdot e^{‐k(t‐t_{0}‐\min TT)} & t\geq t_{0}+\min TT \end{array}\right)$$

where F is tissue perfusion, t_0_ is bolus arrival time, minTT is minimal transit time, E is extraction fraction, and k is excretion rate. Perfusion mapping requires implementation of the model fitting for every muscle voxel in the field of view. For such analysis, we ran the optimization with one set of initial values for the IRF parameters: F 350 ml min^−1^100g^−1^, t_0_ 2 s, minTT 6 s, E 0.4, and k 0.15 s^‐1^. In this study, the “regular fit” took about 3–5 min to generate one perfusion map of matrix size 128 × 128. A schematic diagram of processing DCE MRI for perfusion mapping is shown in Figure 1, and more details of the procedure can be found in previous studies (Zhang et al., 2019). To avoid the potential misfit problem, we also performed the model fitting with a multigrid method, or “multi‐grid fit.” Specifically, 25 runs of optimization were independently initialized with different sets of parameter values, with all the combination of 5 *F* values (100, 150, 200, 250, 300 ml min^−1^100 g^−1^) and 5 t_0_ values (2,3,4,5,6 s). The fitted parameter values from the optimization run with the lowest fitting residue (among the 25 runs) were regarded as the optimal values. Our preliminary study suggested that initializing optimization with multiple values for F and t_0_ would largely avoid the misfit problem for parameter F. The multigrid fit takes 25 times of processing time as in the regular fit, that is, about 70–80 min for one perfusion map of matrix size 128 × 128. The perfusion estimates from the multigrid fit were used as reference perfusion values for both NN training and testing evaluation. All the model fittings in this study were performed on a personal computer (Windows 7, Intel Core i7@2.8GHz, RAM 4.0 GB) using Matlab‐based in‐house programs.

### 2.3 Training of NN for quantifying muscle perfusion

We performed the training and testing of NN in TensorFlow (version 1.13; Python 3.7.3, Spyder 3.3.4, Anaconda Navigator environment) (Abadi, Barham, & Chen, 2016). As the focus of this study was on the training data, we chose a neural network with a simple architecture, and most of the settings were empirically determined in a previous study (Conlin, Li, & Decker, 2019). A fully connected feed‐forward neural network was used, with seven hidden layers and 70 nodes per layer for a good balance between implementation speed and outcome accuracy for our application. Nonlinearity was introduced at each node via the rectified linear unit activation function. The network was compiled with the “adam” optimizer and the “sparse_categorical_crossentropy” loss. The input for the network was an 80‐element vector that concatenated 40 TC points and 40 AIF points for a tissue voxel, and the output was a 40‐element vector that contained probabilities for 40 discretized perfusion values ranging from 10 to 400 ml min^−1^100 g^−1^. The optimal perfusion value of the tissue voxel would be the one with the highest probability value. We chose to use this discretized‐output approach for its potentially higher efficiency and slightly higher accuracy as compared to training with continuous perfusion values in a preliminary study. The NN was trained for 20 epochs using the default learning rate of 0.001 and batch size of 32. For the NN training, perfusion values from multigrid fit were used as reference values.

To explore the optimal NN training strategy, we trained the network with different sets of data, and then compared the trained networks on a same group of testing data. First, the network was trained by the 20 human datasets of the “diverse group” (Table 1), using the perfusion estimates from multigrid model fit. This group contained 88,154 tissue voxels. Second, the network was trained by the 20 human datasets of the “homogeneous group” (10 young healthy subjects, stimulated by 8‐lb exercise only). This group contained 68,933 voxels. Third, the network was trained by the combined 40 datasets. Fourth, the network was trained by the 20 datasets from the diverse‐group data, but used perfusion estimates from regular fit as reference values (less accurate as those from multigrid fit). All the trained networks were tested on the remaining eight datasets of the diverse group (Table 1), which included 25,313 voxels. For clarity, in the following of the paper we will denote perfusion estimates by the multigrid fit as F_0_, those by the regular fit as F_1_, and the estimates by the above NN methods as N_1_, N_2_, N_3,_ and N_4_, respectively.

### 2.4 Comparison of the differently trained networks

For the eight testing datasets, the perfusion estimates by multigrid fit (F_0_) were regarded as reference values for assessing the accuracy of the other estimates, including those by regular fit (F_1_) and by NN of various training (N_1‐4_). For the estimates by each method, mean, standard deviation (*SD*), and median were computed. To quantify the error in each method's perfusion estimates, we computed mean error, root mean square error (RMSE), and mean absolute error (MAE) of the estimates as compared to their corresponding F_0_ values. We investigated if the estimates by the NN methods (N_1‐4_) were more accurate than those by regular fit (F_1_), and if the estimates by NN trained with diverse group (N_1_) were more accurate than N_2‐4_, using paired *t* tests. P value of less than 0.05 was regarded as significant.

## 3 RESULTS

Table 2 summarizes the perfusion estimates for the eight testing data by the different methods. Compared to the reference values F_0_, the estimates by regular model fitting (F_1_) had RMSE of 37.5 ml min^−1^100 g^−1^, MAE of 22.3 ml min^−1^ 100 g^−1^, and correlation coefficient R of 0.889. The NN trained by the diverse group (N_1_) performed significantly better with lower difference (RMSE 26.0 and MAE 15.9; *P* value < .001) and higher correlation (R 0.949).

**Table 2:** Perfusion estimates for the eight testing data by the different methods

	F_0_	F_1_	N_1_	N_2_	N_3_	N_4_
Mean	103.8	102.3	102	90	100	109
*SD*	81.8	74.7	80	62	78	77
Median	72.4	78.6	70	80	70	80

		F_1_‐F_0_	N_1_‐F_0_	N_2_‐F_0_	N_3_‐F_0_	N_4_‐F_0_
Mean	‐	−1.5	−1.5	−13.8	−3.6	3.7
RMSE	‐	37.5	26.0	43.8	27.0	33.5
MAE	‐	22.3	15.9 **^&^**	24.9 **^#^**	15.8 **^&^**	23.5 **^#^**
Correlation	‐	0.889	0.949	0.850	0.944	0.912

Abbreviations: F_0_, values from multigrid model fitting; F_1_, regular model fitting; MAE, mean absolute error; N_1_, 20 diverse data; N_1‐4_, estimates from NN trained by different strategies; N_2_, 20 homogeneous data; N_3_, 40 combined data; N_4_, 20 diverse data with regular fit as reference; RMSE, root means square error; *SD*, standard deviation. Superscript “&” indicates that MAE was significantly lower than that of F_1_. Superscript “#” indicates that MAE was significantly higher than that of N_1_. Unite for all values except for correlation: ml min^−1^ 100 g^−1.^

When trained with the homogeneous group, the network gave estimates (N_2_) with error significantly higher than N_1_ or F_1_ (*P* value < .001). The inclusion of the 20 homogeneous data in the 20 diverse data for NN training resulted in perfusion estimates of comparable accuracy (N_3_ versus. N_1_). Using the regular‐fit values as the reference for NN training, the trained network (N_4_) gave estimates with accuracy comparable to the regular‐fit estimates F_1_ and significantly lower than N_1_ (*P* value < .001).

**Figure 2:**
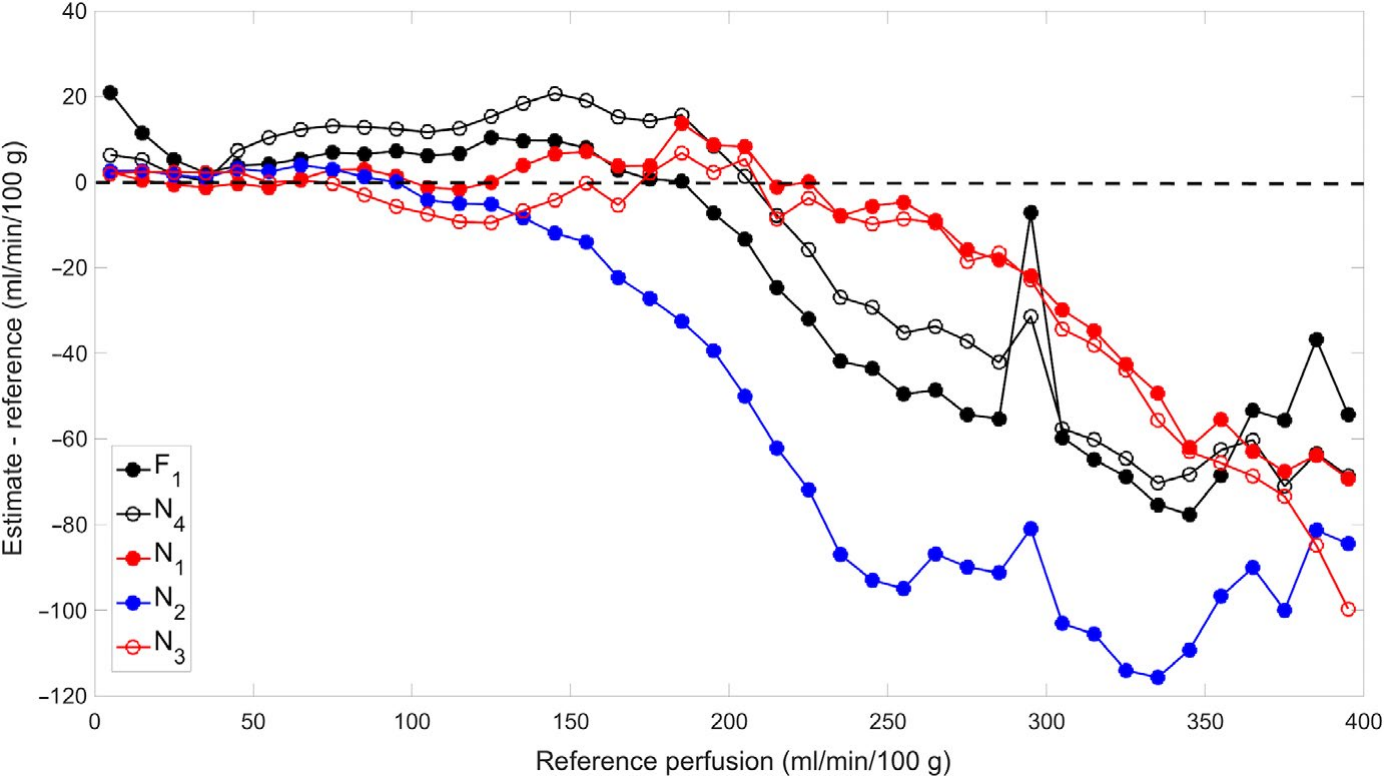
Estimation error of the different methods, over the range of reference perfusion. Along the X axis, the reference values were discretized by intervals of 10 ml min^−1^ 100 g^−1^. For all reference values in each interval, the errors of perfusion estimates by a method were averaged and set as the Y‐axis value of the plotted point. The low‐error spike near to perfusion of 300 ml min^−1^ 100 g^−1^ in the F_1_ (regular model fitting) curve was due to misfit for a group of voxels by model fitting. For these voxels, optimization of both the multigrid fit and regular model fit left the parameter of perfusion unchanged at its initial values of 300 and 350 respectively. This overestimation by F_1_ at 300 reduced its averaged error over the perfusion interval of 290–300 ml min^−1^ 100 g^−1^. F_1_ and N_1‐4_ denotes the different methods as specified in the caption of Table 2

As a further comparison of the estimation errors, we plotted the perfusion error against the reference perfusion (Figure 2). Regular model fitting (F_1_) estimated with error less than 10 ml min^−1^100 g^−1^ for perfusion lower than around 150 ml min^−1^100 g^−1^, but for higher perfusion values, the error increased. The NN trained with regular fit as reference (N_4_) had a similar pattern of error as F_1_. The NN trained by the diverse group (N_1_) maintained low estimation error for perfusion values lower than 250 ml min^−1^ 100 g^−1^, significantly better than F_1_. The NN trained by the 40 datasets (N_3_) shows almost an identical pattern to N_1_. The NN trained by the 20 homogeneous data (N_2_) had a similar pattern, but its error started to increase at perfusion of 100–150 ml min^−1^100 g^−1^ and to a much higher level than that of N_1_ or N_3_.

**Figure 3:**
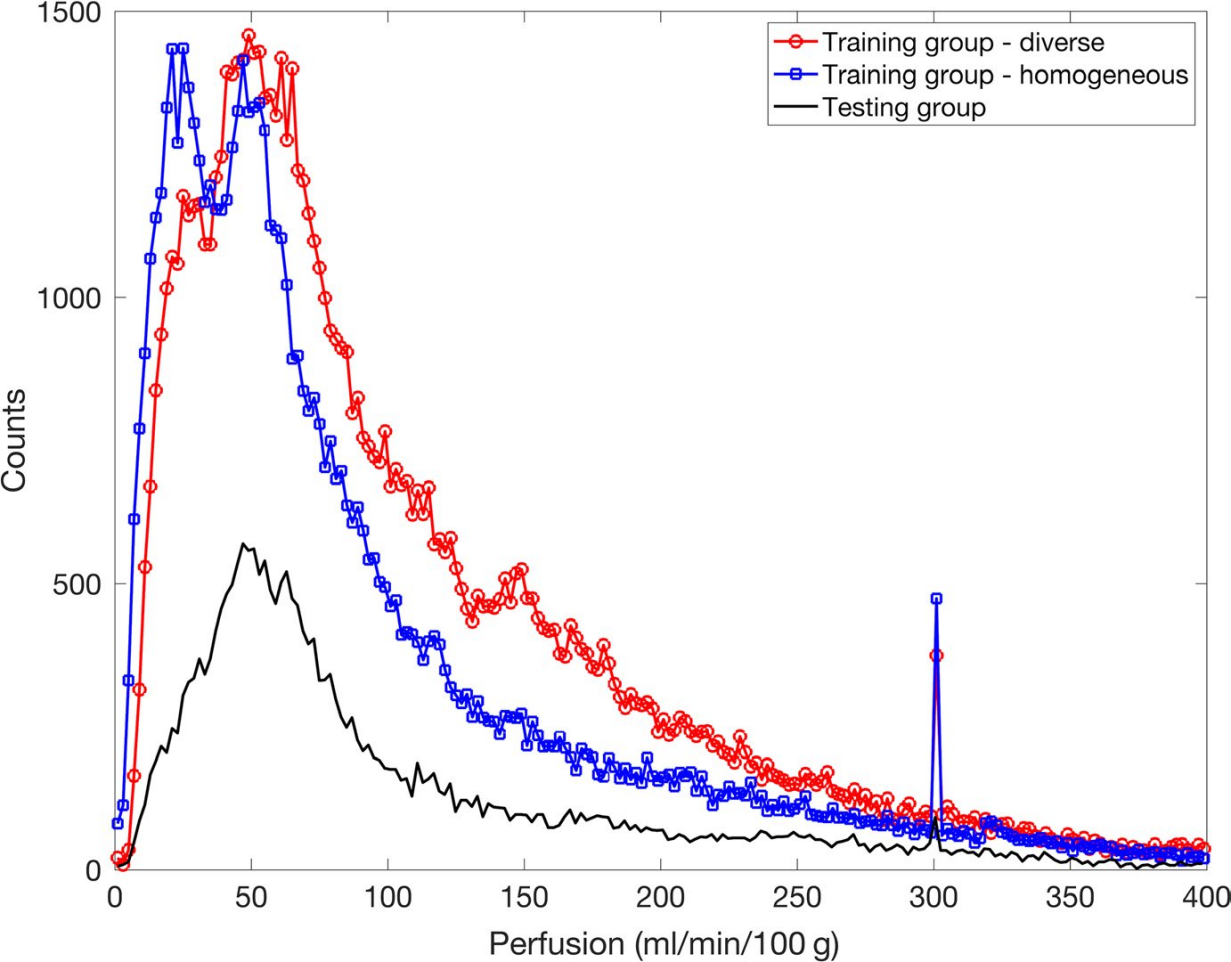
Histogram of perfusion values in the training and testing groups. Median and mean for the diverse training group (20 datasets) were 80.8 and 106.6, for the homogeneous training group (20 datasets) were 62.8 and 91.9, and for the testing group (eight datasets) were 72.4 and 103.8 ml min^−1^ 100 g^−1^. The spikes at perfusion value of 300 ml min^−1 ^100 g^−1^ were because for multiple voxels presumably with high perfusion, the model fitting ended at a local optimum with the initially chosen perfusion value unchanged at 300

To partially explain the nonuniform estimation error of the NN methods across the perfusion range, we plotted the histogram of the reference perfusion values of both the diverse and homogeneous training groups (Figure 3). For both the groups, majority of the values were less than 200 ml min^−1^ 100 g^−1^. The diverse group had more voxels with high perfusion values than the homogeneous group (mean value 106.6 versus. 91.9 ml min^−1^ 100 g^−1^). The distribution of perfusion values in the testing group (mean value 103.8 ml min^−1^ 100 g^−1^) was comparable to that of the diverse group.

**Figure 4:**
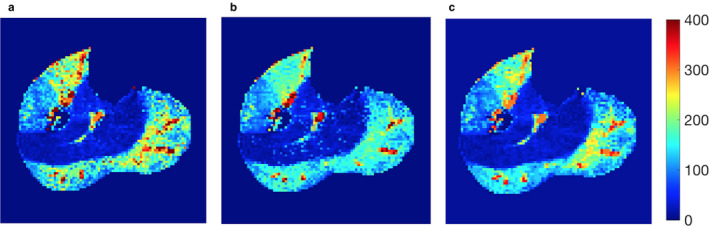
Perfusion maps generated by the various methods for a same subject data. (a) Perfusion map by the multigrid model fitting (F_0_); (b) regular model fitting (F_1_); (c) NN trained by 20 diverse data (N_1_). All the maps were cropped to remove most background regions

The performance of the methods was also demonstrated by the generated perfusion maps for a same dataset. The data was acquired from a young healthy subject stimulated with 8‐lb plantar flexion for 3 min. Figure 4 displays the perfusion maps by multigrid fit (F_0_), regular fit (F_1_), and NN trained by diverse data (N_1_). To generate the map, the multigrid fit took 79 min 34 s, the regular fit 2 min 41 s, and the NN methods each took less than 1 s. All the activated muscle groups, including medial and lateral gastrocnemius and anterior tibial muscles, and even the arterial regions inside the muscles were correctly highlighted in the map generated by the NN method.

## 4 DISCUSSION

Mapping muscle hyperemia with DCE MRI is a promising tool for assessing muscle function and performance, but the voxel‐wise quantification with numerical optimization is time consuming, preventing its real‐time implementation. In this study, we tested the feasibility of using NN to estimate muscle perfusion from DCE MRI data. With the NN method, perfusion maps of eight testing datasets (about 25,000 voxels) were generated almost instantaneously, and the estimates were comparable to the values from the multigrid model fit that took about 70–80 min for each dataset (one slice of image matrix 128 × 128). Our comparison of the various training strategies indicated the importance of properly preparing training data for NN.

One popular application of artificial neural network in medical imaging is to emulate human expert's capability in performing some sophisticated processing procedures, for example,. segmentation in noisy images or classifying abnormalities. We propose a new application to compute tissue perfusion, and use the neural network to circumvent the ordinary approach of computationally intensive deconvolution. A typical two‐dimensional map contains 1000–3000 voxels, and numerical optimization for each voxel makes the mapping process prohibitively slow. Perfusion mapping for exercise‐stimulated muscles is even more challenging than for other organs, because muscle perfusion could vary within a large range with the stimulation. This study shows that with the NN approach a perfusion map can be generated rapidly with relatively high accuracy, potentially enabling real‐time mapping of tissue perfusion during an MRI scan. Real‐time perfusion mapping is obviously invaluable for emergency imaging, such as evaluation of acute stroke. For exercise‐stimulated muscle imaging, immediate perfusion mapping for a pilot scan reveals magnitude and spatial distribution of the stimulated hyperemia, which can be used to personalize the excise stimulation for the following scans in the same exam.

Proper training of NN is important to achieve optimal performance of the network. The first factor we considered in this study was the heterogeneity of the training data. The training data from the diverse group included three types of subjects: young healthy, elderly healthy, and PAD, and each subject was stimulated by exercise of at least two different intensities. Based on an established relationship between cardiac output and AIF (Zhang et al., 2009), an increase of cardiac output with exercise would lead to a proportional decrease in the area under the first pass of AIF. In addition, within field of view of imaging different muscle groups could respond to exercise stimulation by very different degrees. Also, perfusion can rapidly increase from almost zero to more than 300 ml min^−1^ 100 g^−1^. These multiple layers of variability would be reflected in the magnitude and shape of the measured AIF and TC. In contrast, the homogeneous group included only 20 young healthy subjects, stimulated by the 8‐lb exercise only. The perfusion histograms in Figure 3 show the more voxels with high perfusion values in the diverse group and in the homogeneous group. This may explain the substantially better performance of the diverse‐group NN in estimating high perfusion values (Figure 2, N1 versus. N_2_). This result indicates that to estimate tissue perfusion accurately with the NN method, training data should match to those of the target data, and the more diverse the collection of the training data, the more robust the network.

Besides AIF and TC, the other input for NN training is the reference value of muscle perfusion. We trained the network using the same diverse‐group data but different reference values—the multi‐grid‐fit and regular fit values. We found that the network trained with the regular‐fit values (N_4_) performed almost identically to regular model fit, and but not to the multi‐grid model fit. This result is not surprising and suggests that it is worthwhile investing more effort to get more accurate reference values for the training data. In our case, the multigrid model fit took 25—30 times longer processing time than the regular model fit (about 75 versus. 3 min for one 128 × 128 map), but the time‐consuming processing needs only to be done once.

This study has multiple limitations. First, we used only one type of NN, while there are numerous types of NN that could be more powerful, such as convolutional neural networks. Nevertheless, our study does show the promise of the NN approach in quantifying DCE MRI perfusion data of stimulated muscles. Second, we performed the perfusion mapping voxel‐by‐voxel, so that the different voxels were processed independently. This approach ignored potential association between neighboring voxels. Future work should explore utilizing the intervoxel association. Third, the thousands of voxels from each dataset were not fully independent. For each dataset, a common AIF was shared by all the voxels, and there may be other physiologic correlations between the different voxels or different muscle groups (Zhang et al., 2019).

In conclusion, the NN method is promising in quantifying DCE MRI data for muscle perfusion. The method is capable of providing perfusion estimates with comparable accuracy as conventional model fitting, and its extremely fast implementation would make real‐time perfusion mapping possible.

## CONFLICT OF INTEREST

The authors declare no conflict of interest.

## AUTHOR CONTRIBUTION

J.L.Z, G.L. and V.S.L. conceived and designed the study, C.C.C., X.L., and G.L. acquired the human DCE MRI data, X.L., C.C.C., and J.L.Z. designed and implemented the deep learning method, K.C. and J.K. provided guidance on fine tuning of the deep learning method, J.L.Z drafted the manuscript, and all authors edited and approved the manuscript.

## ETHICAL STATEMENT

This study was approved by Institutional Review Board (IRB) of University of Utah (IRB # 00,100,752), and the subjects signed written consent form before the experiment.
